# Wide-field imaging of sickle retinopathy

**DOI:** 10.1186/s40942-019-0177-8

**Published:** 2019-12-12

**Authors:** Marguerite O. Linz, Adrienne W. Scott

**Affiliations:** Retina Division, Wilmer Eye Institute, Johns Hopkins University School of Medicine, 600 North Wolfe Street, Maumenee 719, Baltimore, MD 21287 USA

**Keywords:** Wide-field imaging, Sickle cell retinopathy, Proliferative, 7-standard fields, Fluorescein angiography, Fundus photography, Sickle cell disease, Ultra wide-field imaging, UWF-FA, UWFF

## Abstract

**Background:**

Wide-field imaging is a newer retinal imaging technology, capturing up to 200 degrees of the retina in a single photograph. Individuals with sickle cell retinopathy commonly exhibit peripheral retinal ischemia. Patients with proliferative sickle cell retinopathy develop pathologic retinal neovascularization of the peripheral retina which may progress into sight-threatening sequelae of vitreous hemorrhage and/or retinal detachment. The purpose of this review is to provide an overview of current and future applications of wide-field retinal imaging for sickle cell retinopathy, and recommend indications for best use.

**Main body:**

There are several advantages to wide-field imaging in the clinical management of sickle cell disease patients. Retrospective and prospective studies support the success of wide-field imaging in detecting more sickle cell induced retinal microvascular abnormalities than traditional non-wide-field imaging. Clinicians can easily capture a greater extent of the retinal periphery in a patient’s clinical baseline imaging to follow the changes at an earlier point and determine the rate of progression over time. Wide-field imaging minimizes patient and photographer burden, necessitating less photos and technical skill to capture the peripheral retina. Minimizing the number of necessary images can be especially helpful for pediatric patients with sickle cell retinopathy. Wide-field imaging has already been successful in identifying new biomarkers and risk factors for the development of proliferative sickle cell retinopathy. While these advantages should be considered, clinicians need to perform a careful risk–benefit analysis before ordering this test. Although wide-field fluorescein angiography successfully detects additional pathologic abnormalities compared to traditional imaging, a recent research study suggests that peripheral changes differentially detected by wide-field imaging may not change clinical management for most sickle cell patients.

**Conclusions:**

While wide-field imaging may not carry a clinically significant direct benefit to all patients, it shows future promise in expanding our knowledge of sickle cell retinopathy. Clinicians may monitor peripheral retinal pathology such as retinal ischemia and retinal neovascularization over progressive time points, and use sequential wide-field retinal images to monitor response to treatment. Future applications for wide-field imaging may include providing data to facilitate machine learning, and potential use in tele-ophthalmology screening for proliferative sickle retinopathy.

## Background

**Fig. 1:**
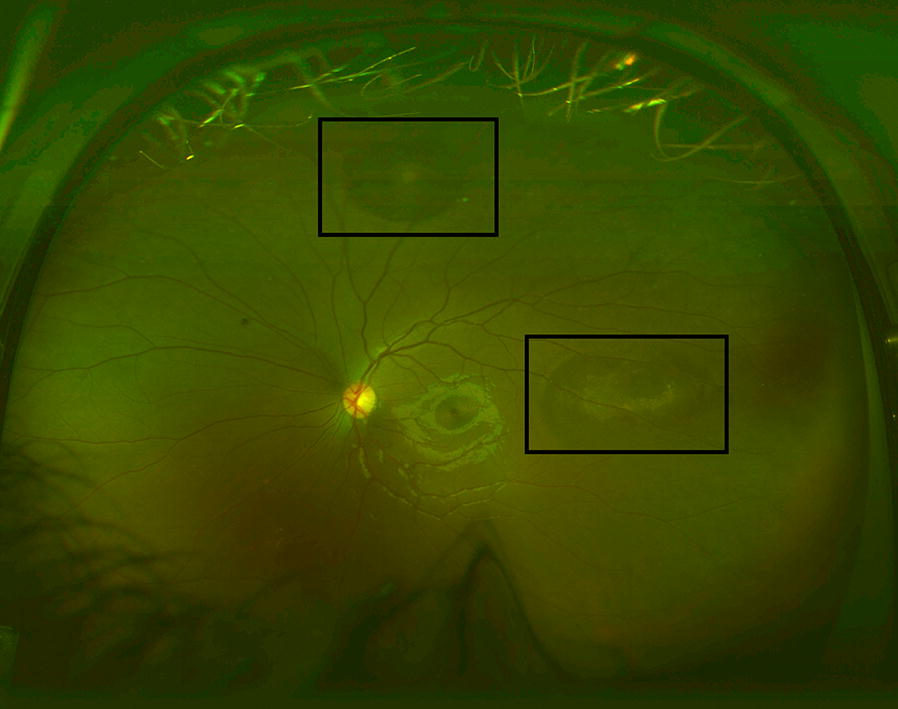
Representative photo of refractile spots, enclosed by black boxes, on ultra wide-field fundus photography. Left eye of a 12-year-old male with Hemoglobin SS sickle cell disease

Sickle cell disease (SCD), the most prevalent genetic blood disorder in the United States, distorts hemoglobin molecules into a “sickled” shape. Vaso-occlusive, hypoxia-inducing events can happen in any organ of the body, including the eye. Ocular manifestations of SCD depend on a variety of factors, including sickle cell genotype. Signs of nonproliferative sickle cell retinopathy observed on clinical examination can include black sunburst lesions (5.6–46% Hemoglobin SS; 20–63% Hemoglobin SC) [1–5], refractile or iridescent spots (3.7–10.6% Hemoglobin SS; 6.7–28% Hemoglobin SC) [1–3] (Fig. 1), and salmon patch retinal hemorrhages (0–3.5% Hemoglobin SS; 4.6–17% Hemoglobin SC) [1, 3, 5]. Retinal imaging detects more subtle structural changes such as macular thinning seen on optical coherence tomography (OCT), as well as areas of peripheral retinal vascular dropout and arteriovenous anastomoses well-visualized on fluorescein angiography (FA).

**Fig. 2:**
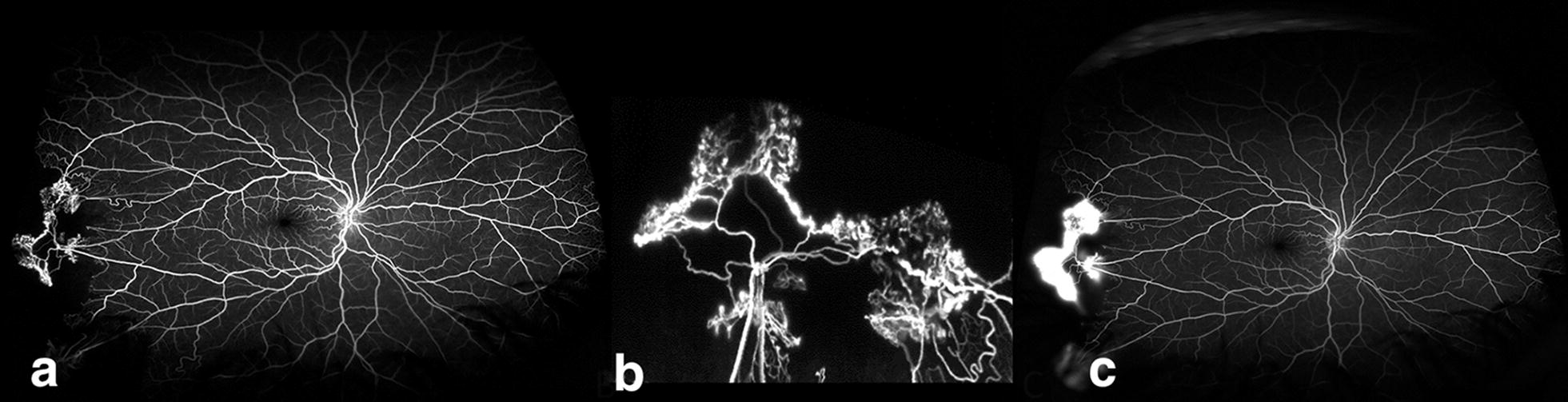
Representative ultra wide-field fluorescein angiogram depicting sea fan neovascularization. Neovascularization is surrounded by an area of non-perfusion in the temporal periphery (**a**) in early frames (00:27:530). **b** Lacy, frond-like vessels are shown in the magnified image of the sea-fan neovascular complex. **c** Late frames show leakage of the neovascularization (01:42:468)

**Fig. 3:**
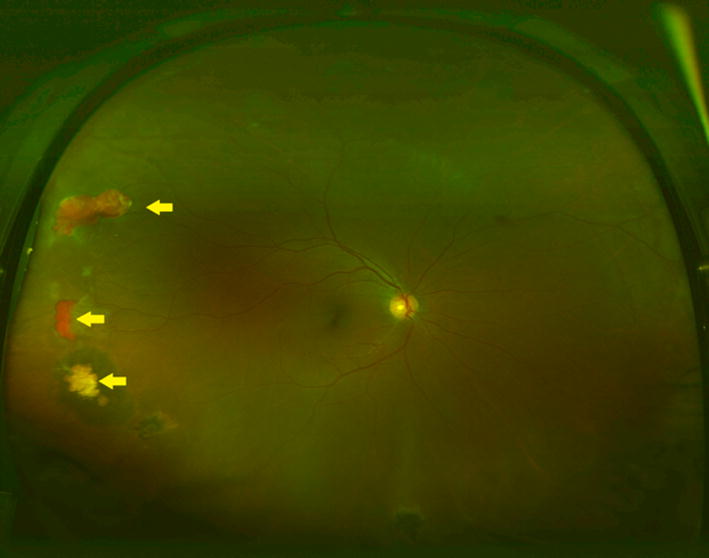
Representative ultra wide-field fundus photograph shows multiple partially vascularized sea fan lesions (yellow arrows)

Retinal hypoxia and the resulting tissue ischemia caused by SCD can stimulate the production of pro-angiogenic factors such as vascular endothelial growth factor (VEGF) which in turn, can stimulate pathologic blood vessel proliferation [6, 7]. Proliferative sickle cell retinopathy (PSR) is a rare, but potentially vision-threatening complication of sickle cell disease. All SCD genotypes can develop proliferative sickle cell retinopathy. Hemoglobin SS patients tend to be more affected with systemic complications such as stroke and painful occlusive crises, but are less likely to develop advanced-stage retinopathy than patients with Hemoglobin SC and other variant genotypes. PSR is reported to affect 25.3–75.9% of Hemoglobin SC patients and 3.5–30.2% of Hemoglobin SS patients [1, 8–12]. Individuals with PSR experience pathologic retinal neovascularization, characteristically in a “sea-fan” shape (Figs. 2, 3), increasing their vulnerability to vitreous hemorrhage and retinal detachment. One of the challenges in caring for patients with sickle cell disease is predicting those patients who will experience retinopathy progression. Hemoglobin SC disease and increasing age are well-supported risk factors for retinopathy [11], but PSR has been reported in patients as young as eight and in all genotypes [10]. Expert consensus recommends regular retinopathy surveillance exams for all individuals with sickle cell disease starting at age 10 in order to identify those at risk for vision loss [13]. Peripheral retinal vascular changes can be subtle based on clinical exam alone. However, at the present time, there are no evidence based guidelines or even expert consensus recommendations on the optimal imaging, if any, by which to evaluate and classify retinopathy in SCD patients.

Although significant macular changes have been identified in SCD patients, sickle cell retinopathy is historically classified by its peripheral retinal manifestations. Vascular remodeling and pathologic neovascularization tend to start in the far periphery. Fundus photography, commercialized in 1926, first achieved a 20-degree field and later upgraded to the standard 30-degree field [14]. The addition of fluorescein dye to fundus photography, fluorescein angiography (FA), proved invaluable to ophthalmologists studying sickle cell retinopathy. FA became the gold standard for surveillance of the retinal vascular changes commonly noted in sickle cell retinopathy. However, these 30-degree photos limited view of the periphery. A protocol developed by the Early Treatment Diabetic Retinopathy Study (ETDRS) Research Group increased the maximum field of view of FA to 75-degrees, by montaging of seven 30-degree photographs (7-standard field photographs) [15].

**Fig. 4:**
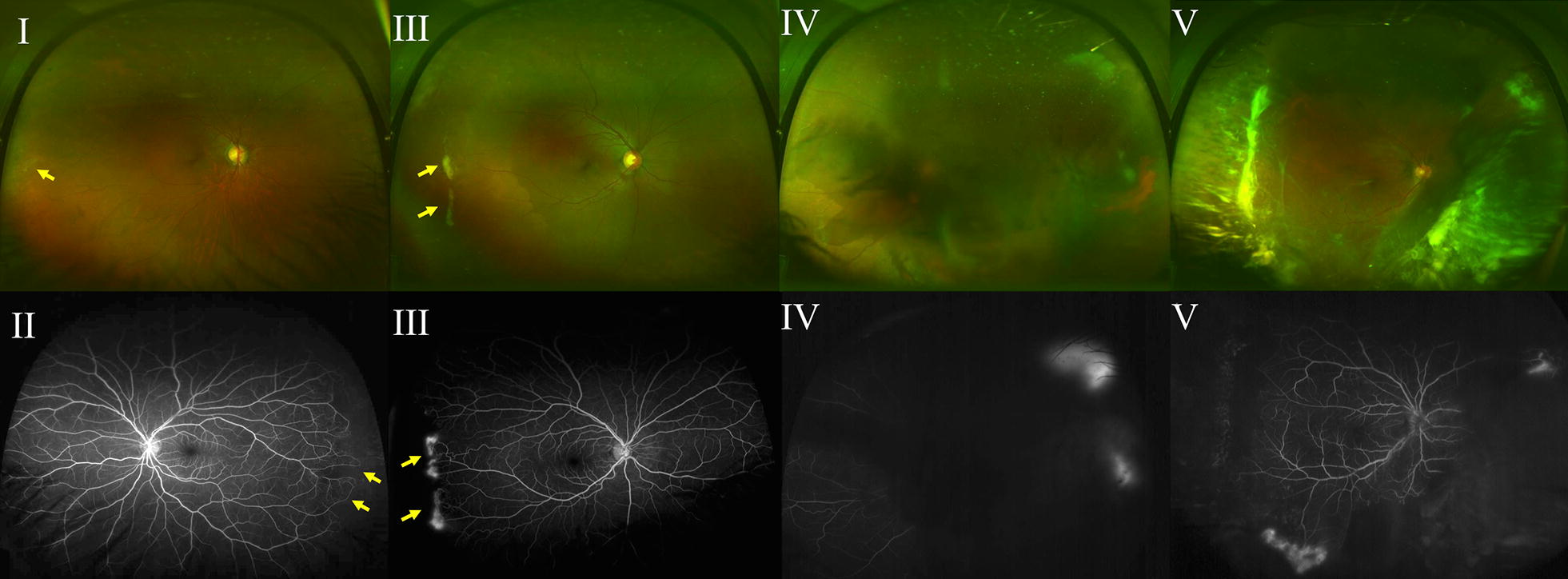
Goldberg stages of sickle cell retinopathy (I–V). **I** Arteriolar occlusion can be observed in the periphery (yellow arrows) of this ultra wide-field fundus photograph. **II** Peripheral arteriovenous anastomoses (yellow arrows) are apparent on ultra wide-field fluorescein angiography. **III** Pathologic neovascularization (yellow arrows) is observed in the periphery of the retina on (top) ultra wide-field fundus photography and (bottom) ultra wide-field fluorescein angiography. **IV** The view of the retina on (top) ultra wide-field fundus photography and (bottom) ultra wide-field fluorescein angiography (bottom) is obscured by a vitreous hemorrhage. Leakage from sea fan neovascularization is observed despite blocking of the fundus view by vitreous hemorrhage. **V** Tractional retinal detachment is observed in the inferior retinal periphery on (top) ultra wide-field fundus photography and (bottom) ultra wide-field fluorescein angiography (bottom). Vitreous hemorrhage is also present in the retinal periphery

**Fig. 5:**
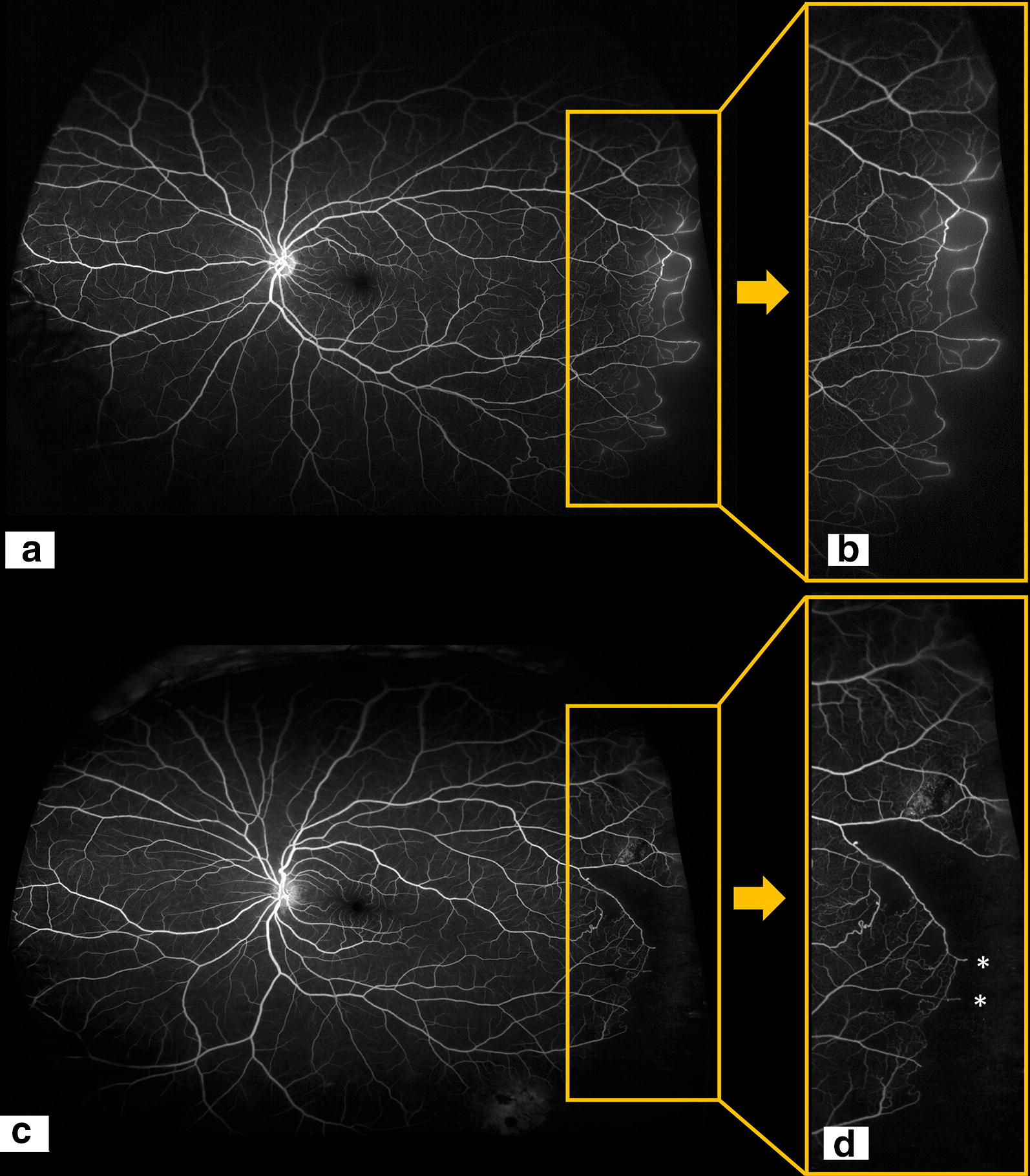
Representative Penman type 1 borders (**a**), highlighted in the inset (**b**). Ultra wide-field fluorescein angiography of a left eye of a 26-year-old female with Hemoglobin SS sickle cell disease (**a** and **b**). The type 1 classification is based on the presence of smooth and continuous looped arteriovenous anastomoses (Penman et al. 1994). Representative Penman type 2 borders (**c**), highlighted in the inset (**d**). Fluorescein angiogram of a left eye of a 33-year-old male with Hemoglobin SS sickle cell disease (**c** and **d**). The type 2 classification is based on the presence of jagged, abnormal arteriovenous anastomoses with capillary buds (asterisks) extending towards the periphery (Penman et al. 1994)

The two most widely used sickle cell retinopathy staging methods were developed based only on features observed on clinical examination and 30-degree standard FA [16, 17]. Reflective of PSR’s classification primarily as a peripheral retinopathy, determination of retinopathy severity specifically relies upon analysis of microvascular abnormalities in the periphery. The Goldberg classification system, developed in 1971, describes five sequential stages of increasing severity: (I) peripheral arteriolar occlusions, (II) arteriovenous anastomoses, (III) neovascularization, (IV) vitreous hemorrhage, and (V) retinal detachment (Fig. 4) [16]. Consequently, in 1994, Alan Penman observed that characteristic peripheral vascular border abnormalities correlated with development of PSR [17]. Penman devised a new staging system based on FA findings classifying PSR based on the relative risk of PSR development [17]. This system classified vascular borders as Type 1 (qualitatively normal, permitting avascular lacunae, hairpin loops, and tortuous vessels) or Type II (abrupt terminations of vessels at the vascular border) (Fig. 5) [17]. In Penman’s description of his classification system, the vascular borders were too far peripheral to allow grading in half of the eyes [17]. Penman stressed that a limitation of the study was that the observations made were incomplete due to the limited view of the retinal periphery using standard FA.

New imaging technologies have emerged since the development of the Goldberg and Penman classification systems, increasing our understanding of sickle cell retinopathy. Two of these technologies, OCT and OCT angiography (OCTA), confirmed the presence of structural macular abnormalities in SCD patients, particularly structural macular thinning most notable in the temporal macular region [18] and loss of macular vessel density [19, 20], respectively. The clinical utility of these macular findings is as yet unknown; therefore, FA remains the primary imaging tool for evaluating retinal perfusion in sickle cell.

**Fig. 6:**
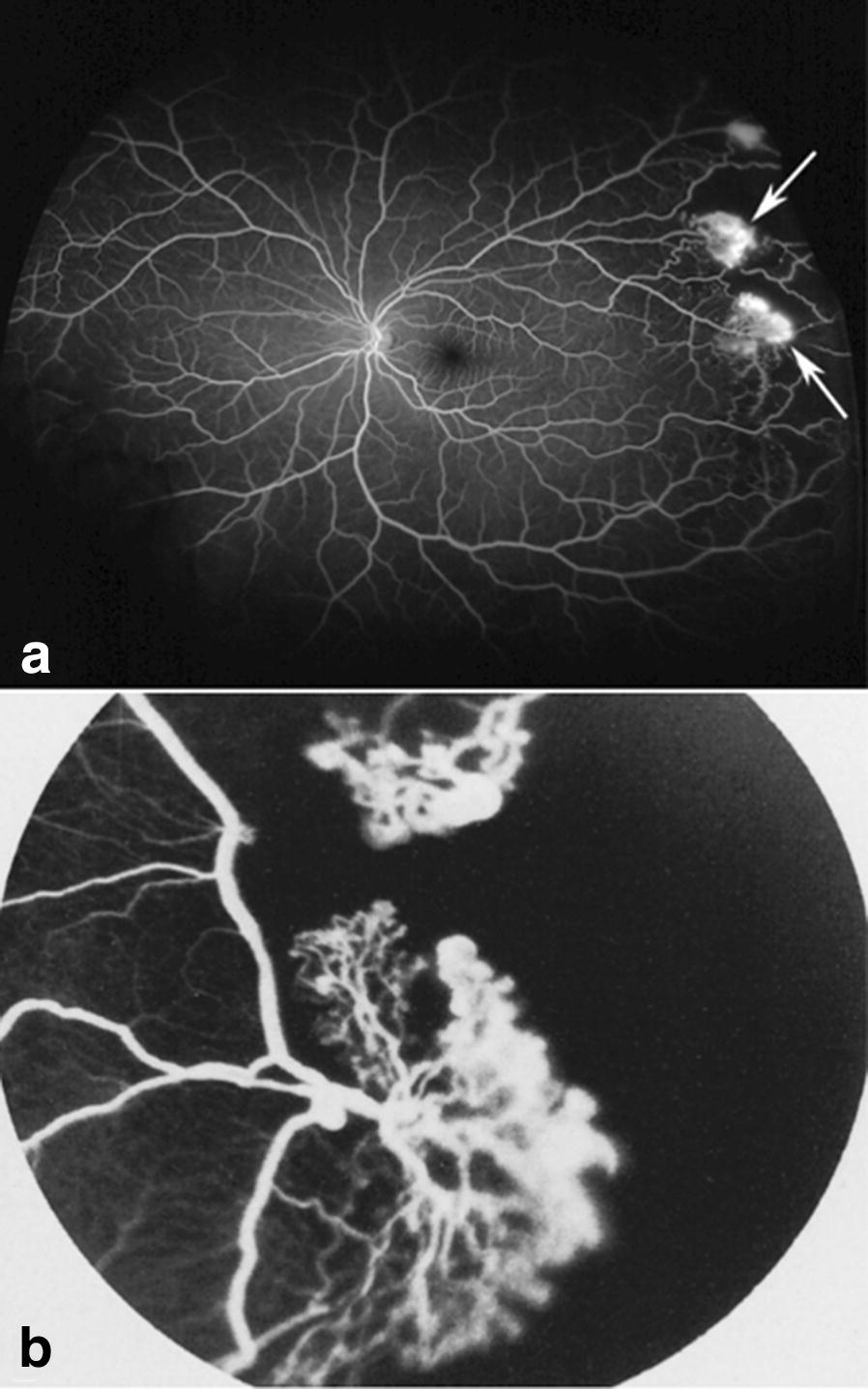
Field of view compared between (**a**) ultra-wide field fundus image and (**b**) 30° image. Both photographs show sea fan lesions in the peripheral fundus of sickle cell disease patients. Reprinted with permission from A.W. Scott, G.A. Lutty, and M.F. Goldberg. Hemoglobinopathies. In: Schachat AP, Wilkinson CP, Hinton DR, Sadda SR, Wiedemann P, eds. *Ryan’s Retina*. 6th Edition. Philadelphia, PA: Elsevier; 2018

**Fig. 7:**
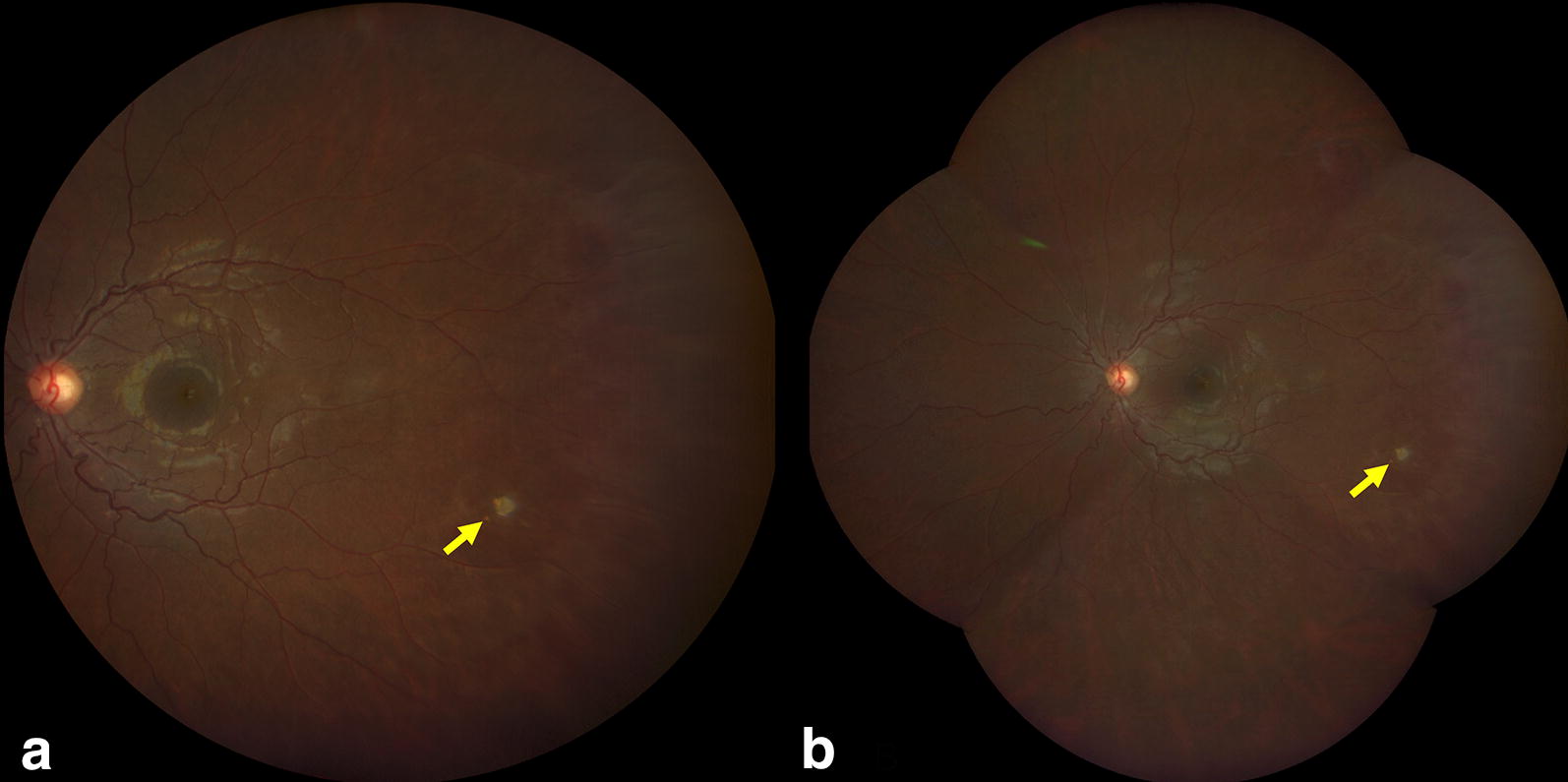
Zeiss Clarus wide-field fundus photography of a Hemoglobin SS sickle cell disease patient. A small chorioretinal scar in the inferotemporal periphery is evident in both (**a**) the 133 degree field single capture and (**b**) the montaged image

In the year 2000, Optos (Dunfermline, Scotland) produced the first commercialized wide-field fundus camera which successfully integrated into clinical practice. This ultra wide-field (UWF) technology expanded the field of view to 200 degrees in one non-steered image, a major advance from the standard 30 degree image (Fig. 6), as well as the 75 degrees covered by the 7-standard field montage. Carl Zeiss, Inc (Oberkochen, Germany) released a non-mydriatic UWF camera (Clarus 500) in 2017 which has the advantage of true color and a maximum field of 267 degrees in a montaged image, but the strong light can be uncomfortable for patients and a single capture is limited to a 133-degree field (Fig. 7). Ultra wide-field fundus photography (UWFF) and ultra wide-field fluorescein angiography (UWF-FA) provide new information about vascular changes occurring in the far retinal periphery. Published reports on the use of UWF imaging in other peripheral retinopathies including diabetic retinopathy, retinal vein occlusion [21, 22], and retinopathy of prematurity [23] support the technology’s clinical utility. For example, when used for diabetic retinopathy, UWF imaging influenced clinical management by detecting increased severity and a greater extent of non-perfusion than standard FA [24], as well as successfully guided targeted pan-retinal photocoagulation laser treatments [25]. Most of the studies evaluating UWF imaging in diabetic retinopathy have been retrospective, but support its utility for identification of peripheral vascular changes. A prospective study led by the Diabetic Retinopathy Clinical Research Network is underway to assess the utility of UWF imaging in diabetic retinopathy. Although research on the use of wide-field imaging in sickle cell retinopathy supports its success in capturing microvascular vascular abnormalities which may be missed by standard imaging in some patients, investigators cautiously advise that this imaging may not always be clinically necessary.

The aim of this review is to assess the utility of wide-field imaging in sickle cell retinopathy and suggest best practices for its use. Moreover, recent research in this field will be presented in this comprehensive review.

## Captures greater severity of disease

Just over a decade after the introduction of UWF imaging, the first report on the utility of UWF-FA to detect PSR was published. This study revealed that UWF-FA identified peripheral microvascular abnormalities undetected by 7-standard field FA in 91.7% (11/12) eyes studied [26]. Furthermore, in 25% (3/12) of eyes, peripheral microvascular abnormalities missed by clinical examination were detected by UWF-FA [26]. This study was limited by its small sample size. The results of this study, however, were supported by a later larger study evaluating 70 eyes of 35 patients with two masked ophthalmologist graders. In this study, both UWF-FF and UWF-FA independently resulted in a higher stage of retinopathy compared to clinical examination [27]. Additionally, UWF-FA resulted in graders assigning a higher Goldberg stage compared to UWF-FF [27]. UWF-FA was able to capture the borders of peripheral vasculature in 98.6% (69/70) of eyes, excluding one eye with a vitreous hemorrhage [27]. Thus, UWF-FA has been shown to be superior in capturing peripheral retinal pathology as compared to data from Penman’s report, in which 7-standard field FA only captured 50% of vascular borders [17].

## Clinical efficiencies of UWF in sickle cell

**Fig. 8:**
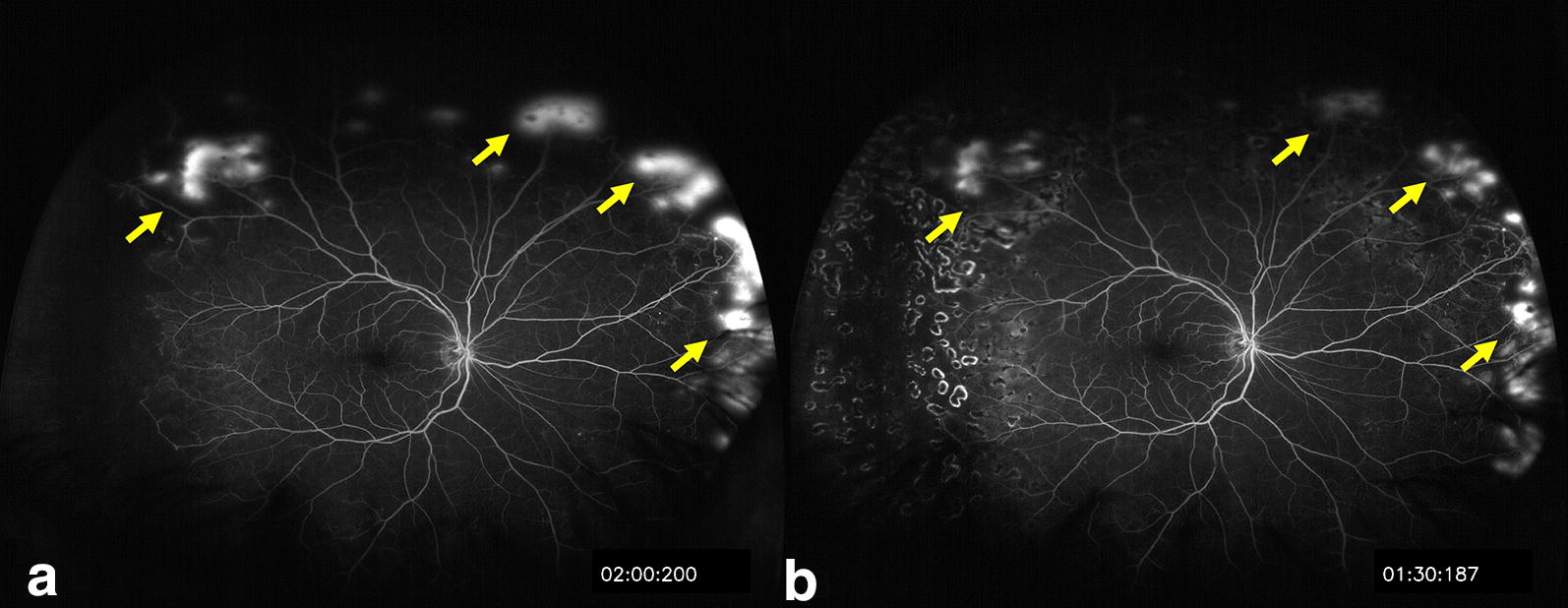
Sixty-seven-year-old male with Hemoglobin SC sickle cell disease at presentation and post- scatter laser treatment. **a** Presentation with multiple sea fan neovascular lesions sequentially imaged with ultra wide-field fluorescein angiography (white arrows). Scatter laser treatment was applied to areas of profound ischemia in the far temporal periphery as well as applied to border of perfused and non perfused retina. **b** 4 months post-presentation and post-treatment, the sea fans show decreased leakage, with no new areas of neovascularization are observed

**Fig. 9:**
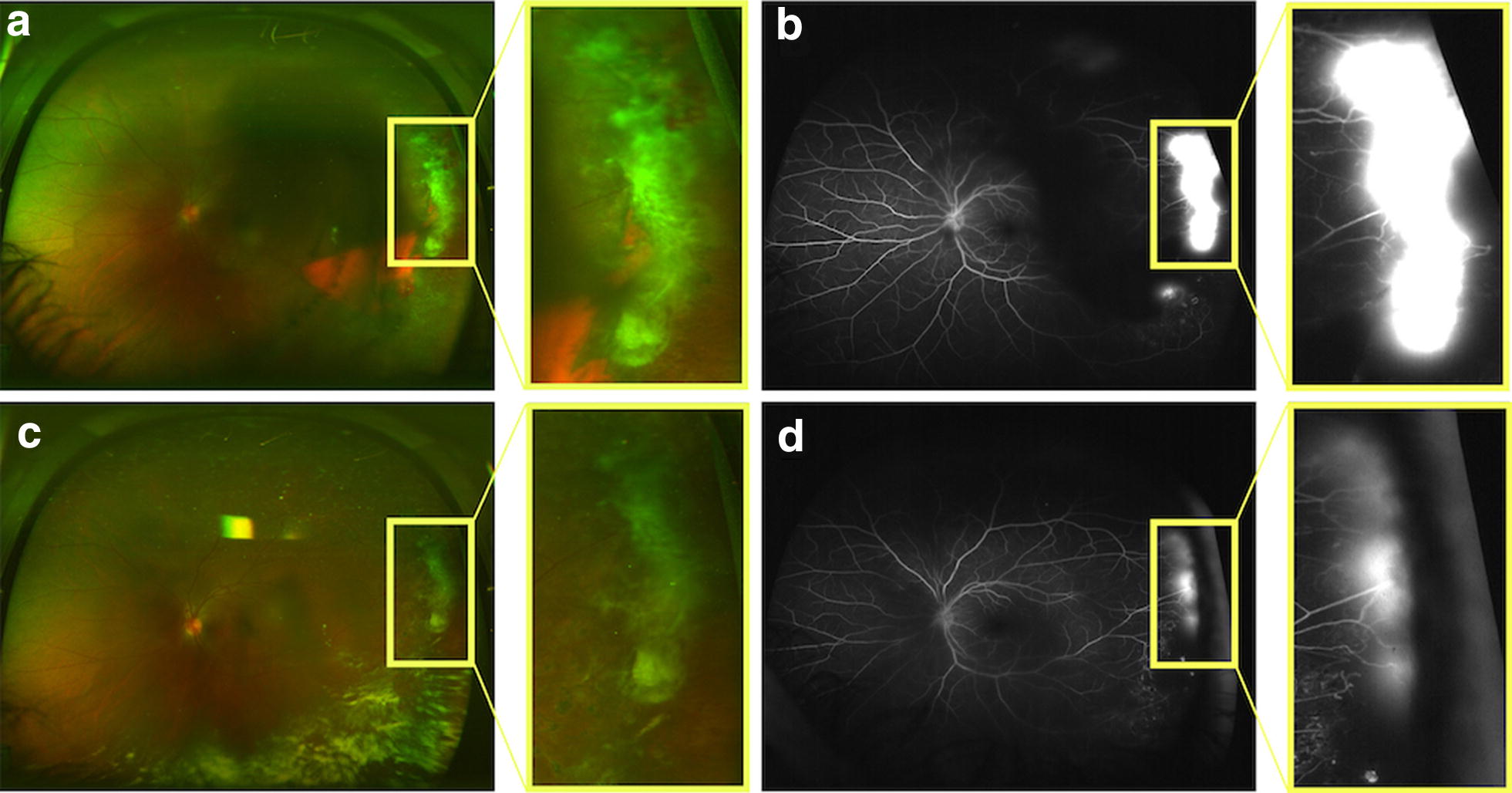
A 33-year-old woman who underwent 2 injections of intravitreal bevacizumab spaced 5 months apart. **a** Ultra wide-field imaging at the initial visit shows peripheral sea fan neovascularization highlighted in the inset that leaks on fluorescein angiography taken at 5 min and 18 s (**b**). After the injections, there is regression of the neovascularization (**c**) with decreased leakage on fluorescein angiography taken at 4 min and 34 s (**d**). Reprinted with permission from Cai CV, Linz MO, Scott AW. Intravitreal bevacizumab for proliferative sickle cell retinopathy: A case series. *Journal of Vitreoretinal Diseases.* 2017; 10.1177/2474126417738627

UWF-FA allows for identification of peripheral retinal vascular abnormalities, and longitudinal UWF-FA allows documentation of the pathology so that any changes can be monitored over time. Neovascular complexes in PSR tend to autoinfarct up to 32% of the time without visual consequence [10]. However, in select cases, neovascular complexes can enlarge, become elevated, and cause progressive traction on the posterior hyaloid and on the retina, resulting in either a tractional or combination tractional-rhegmatogenous retinal detachment. These progressive changes of peripheral neovascularization over time may be documented with UWF imaging, and progression of neovascularization may alert the retina specialist that treatment such as scatter laser photocoagulation (Fig. 8) or anti-VEGF therapy (Fig. 9) should be considered.

Along with benefitting the physician’s ability to monitor disease progression, UWF imaging offers small direct benefits for both patients and photographers. An alternative to UWF imaging is 7-standard-field imaging. This methodology involves taking comparatively more photos and necessitates greater cooperation from the patient. UWF, on the other hand, minimizes the number of photos taken. This convenience can be especially important for sickle cell patients who suffer chronic pain, and for whom long sessions of imaging can be taxing. It also benefits pediatric sickle cell patients who may struggle with focus, agitation with testing, and restlessness. Recent research suggests prevalence of sickle cell retinopathy in children is much higher than previously reported [28]; therefore, it is important that imaging is accessible for young children. Further, for the photographer, UWF imaging requires fewer technical skills than 7-standard field photography.

## Correlations with multimodal imaging and retinopathy risk factors

**Fig. 10:**
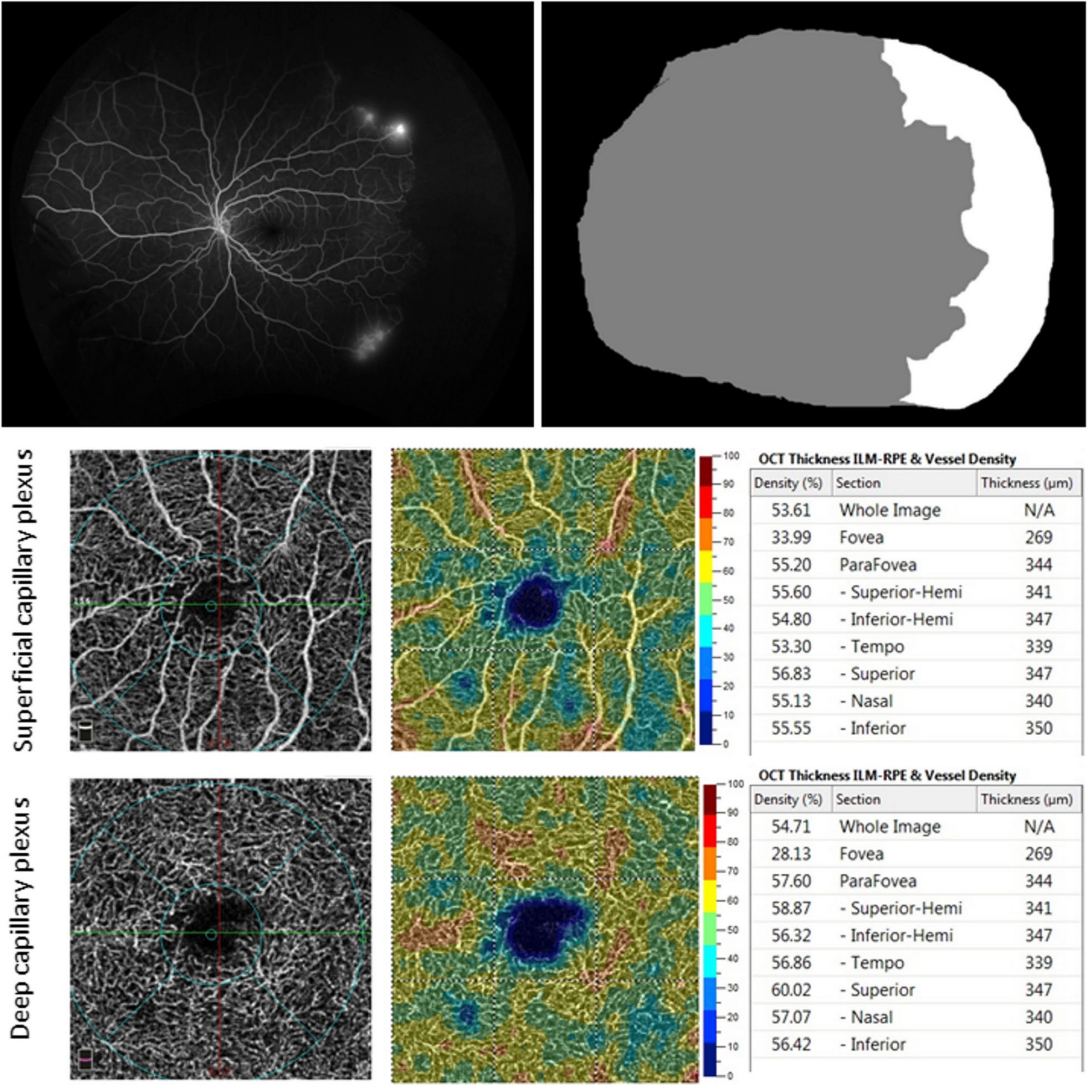
Example images of ischemic index calculation. Left eye of a 27-year-old man with sickle cell SC genotype and Goldberg stage 3 (proliferative) sickle cell retinopathy. Top row, Montage ultra wide-field fluorescein angiography (left). The area of non perfusion is shaded in white on the mask (right), whereas the total perfused area is shown in gray. Middle row, Superficial capillary plexus OCT angiography (left), vessel density map (middle), and vessel density values (right). Bottom row, Deep capillary plexus optical coherence angiography (left), vessel density map (middle), and vessel density values (right). OCTA loss of macular flow was significantly correlated with ischemic index in sickle cell retinopathy patients. Flow loss is commonly noted in both the superficial and deep capillary plexuses, and often more commonly observed in the deep capillary plexus (arrow). RPE = retinal pigment epithelium. Reprinted with permission from Han IC, Linz MO, Liu TYA, Zhang AY, Tian J, Scott AW. Correlation of ultra-widefield fluorescein angiography and OCT angiography in sickle cell retinopathy. *Ophthalmology Retina.* 2018;2(6):599-605

**Fig. 11:**
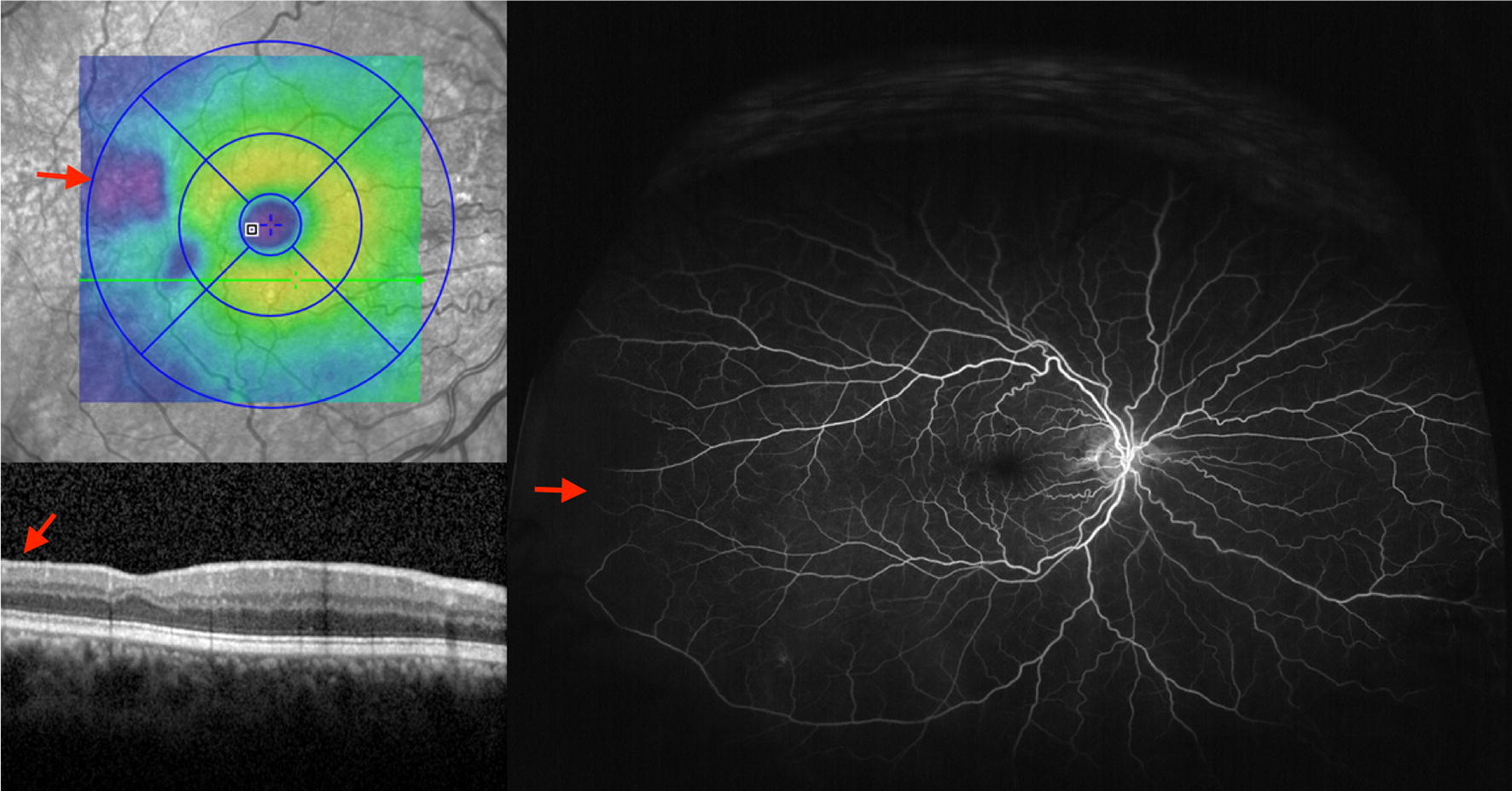
Multimodal retinal imaging of a 58-year-old male with Hemoglobin SS sickle cell disease (Top left). Optical coherence tomography macular density map shows structural thinning (dark blue areas on thickness map, red arrow) correlating with (bottom left) thinning on optical coherence tomography of the macula (red arrow). Ultra wide-field fluorescein angiography of the same eye shows peripheral capillary drop out in the temporal retinal periphery (red arrow)

The ability of UWF-FA to capture a greater extent of the retina including the vascular borders at a single time point and in a single frame, allows for opportune collection of data, including quantification of retinal ischemia. One measurement permitted by UWF-FA is measurement of ischemic index, the percentage of non-perfusion per total visible retina (Fig. 10). Conversion of a 3-dimensional globe into a 2-dimensional image distorts UWF imaging; however, a software developed by Optos minimizes image analysis errors by calculating for stereographic projection distortion and horizontal stretch [29]. Han et al. established a direct, statistically significant correlation between ischemic index and retinopathy severity, supporting the prospect of ischemic index to serve as biomarker for advanced disease [30, 31]. Other statistically significant associations of ischemic index in SCD patients include hemoglobin subtype, age, decreased macular vessel density on OCTA (Fig. 10), and presence of retinal thinning on OCT [30, 31] (Fig. 11). Further studies are needed to support the generalizability of this data. While clinical utility of ischemic index remains unclear, in the future, retinal features such as progressive non perfusion observed with UWF imaging may improve our understanding of structural changes occurring in the retinas of SCD patients, and may help elucidate peripheral retinal characteristics that predict PSR risk.

## Is UWF imaging essential for the diagnosis and monitoring of SCR?

**Fig. 12:**
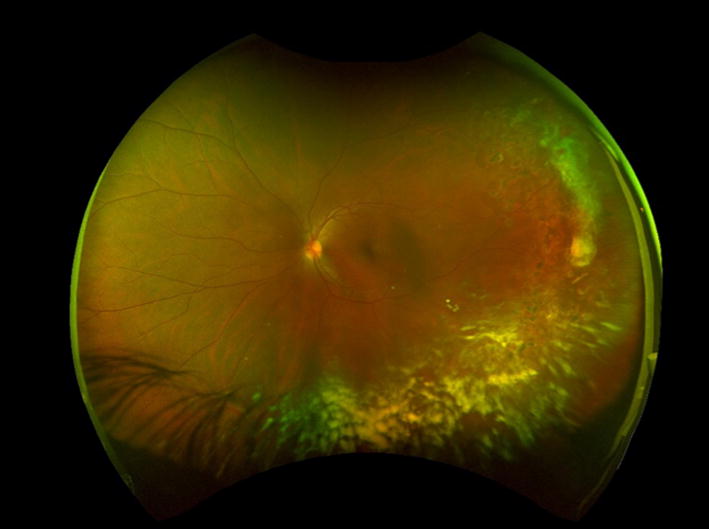
Ultra wide-field fundus photo taken with the Optos Caifornia platform shows peripheral vitreous hemorrhage. Image of the retina of a 34-year-old woman with Hemoglobin sickle beta thalassemia, and vitreous hemorrhage is consistent with Stage IV sickle cell retinopathy

Despite the previously described advantages of UWF imaging, there may not be a clinically significant added benefit for SCD patients over clinical examination. A study by Han et al. included 70 eyes, and showed that UWF-FA did not alter treatment decisions compared to a full exam [27]. Though UWF imaging showed greater severity, severity mostly only increased from Goldberg stages 1 to Goldberg stage 2, both of which are nonproliferative stages for which observation is the preferred management [27]. UWF-FA detected neovascularization otherwise undetected on exam in only one participant, and this neovascular lesion was not deemed severe enough to indicate treatment [27]. The small number of participants with proliferative disease (n = 8) included in this study may bias results, however, even with greater detection of disease, a risk–benefit assessment is necessary. UWF-FA carries the same drawbacks as standard FA, including the time burden on the patient, the cost of the photography and the procedure, and the risks involved with injection of the fluorescein dye. Weaknesses of UWF imaging, specifically, include reduced peripheral resolution, slightly distorted pseudocolor photos, artifacts such as eyelashes or eyelids obscuring the field of view [32], and the expense of the technology. Newer UWF imaging technologies offer improved color and less artifact (Fig. 12).

Notwithstanding its limitations, UWF imaging is clinically worthwhile to perform when examination by a retina specialist suggests significant peripheral pathology, particularly presence of arteriovenous anastomoses or pathologic retinal neovascularization [27]. Because published literature shows that the prevalence of PSR is significantly higher in patients with Hemoglobin SC disease, ophthalmologists should give additional consideration to obtaining UWF imaging these patients [27]. Additional consideration may also be given to patients exhibiting temporal macular atrophy on spectral domain OCT due to its association with neovascularization in sickle cell patients [33]. UWF imaging also may be clinically useful in monitoring treatment response and for instances in which careful fundus examination is not performed.

## Future directions

While UWF imaging may not provide a direct, additional benefit to most sickle cell patients compared to standard fundus and FA imaging, the technology holds great potential to inform classification and surveillance in sickle cell retinopathy, and to guide treatment in PSR. Scatter laser photocoagulation, the most common treatment for severe PSR, has not been proven to be more effective than observation for regression of neovascularization [34]. This highlights the importance of further study of the pathogenesis and natural history of PSR, identification of systemic risk factors associated with PSR, and other PSR treatment options [34]. The rarity of PSR makes recruiting for large-scale prospective clinical trials to determine the best methods of treatment a challenging endeavor. Therefore, any extra data or convenience that imaging technology can provide in monitoring treatment is highly advantageous. Neither the Goldberg nor Penman classification systems have been updated in decades despite advances in technology. The development of UWF imaging, along with other novel retinal imaging devices such as OCTA, provide new insights into sickle cell disease which could inform modernized classification of PSR and new surveillance guidelines.

The incidence of sickle cell disease worldwide is expected to rise significantly over the next few decades [35]. In comparison with 7 standard field photography, the Optos UWF technology permits more efficient imaging because it does not require dilation, and UWF fundus images are high quality and high resolution even when obtained by a trained non-ophthalmic technician [36]. This suggests that UWF technology could advance tele-ophthalmology systems, increasing access to eye care and screening to individuals in medically underserved areas. Another potential future application for UWF imaging would be to feed UWF images into machine learning technology. Recently, deep learning technologies using 30-degree fundus images have proven successful in the detection of diabetic retinopathy [37]. Deep learning algorithms, aided by UWF imaging may one day “learn” to recognize subclinical biomarkers of PSR, so that we could predict progression of retinopathy. Additionally, deep learning software could potentially “learn” to differentiate the presence or absence of retinal sea fan neovascularization, facilitating a novel precision medicine approach to PSR. Automated detection of sickle cell retinopathy and identification of disease severity would expand access to retinopathy surveillance exams in medically underserved areas globally.

## Conclusion

UWF retinal imaging broadens not only our field of view, but also our knowledge of the extent of pathology in both non proliferative sickle cell retinopathy and in PSR. Peripheral pathology in SCD including sea fan neovascular complexes and peripheral retinal ischemia may be identified and imaged with UWF fundus photography or UWF FA to assess disease progression over time. While UWF imaging may not be clinically necessary for all SCD patients, it is an important tool for imaging patients at high risk of PSR and for informing new methods of retinopathy surveillance, monitoring, and management of PSR.

## Data Availability

Not applicable.

## Abbreviations

ETDRS: Early Treatment Diabetic Retinopathy Study
FA: fluorescein angiography
OCT: optical coherence tomography
OCTA: optical coherence tomography angiography
PSR: proliferative sickle cell retinopathy
SCD: sickle cell disease
UWF: ultra wide-field
UWF-FA: ultra wide-field fluorescein angiography
UWFF: ultra wide-field fundus photography
VEGF: vascular endothelial growth factor

## References

[1] Clarkson JG (1992). The ocular manifestations of sickle-cell disease: a prevalence and natural history study. Trans Am Ophthamol Soc..

[2] David RC, Moraes Júnior HV, Rodrigues MP (2011). Ocular and electroretinographic changes in sickle cell disease. Arq Bras Oftalmol..

[3] de Almeida Oliveira DC, Carvalho MO, do Nascimento VM, Villas-Bôas FS, Galvão-Castro B, Goncalves MS (2014). Sickle cell disease retinopathy: characterization among pediatric and teenage patients from northeastern Brazil. Rev Bras Hematol Hemoter..

[4] Friberg TR, Young CM, Milner PF (1986). Incidence of ocular abnormalities in patients with sickle hemoglobinopathies. Ann Ophthalmol..

[5] Osafo-Kwaako A, Kimani K, Ilako D (2011). Ocular manifestations of SCD at the Korle-bu Hospital, Accra, Ghana. Eur J Ophthalmol..

[6] Cao J, Mathews MK, McLeod D, Merges C, Hjelmeland L, Lutty G (1999). Angiogenic factors in human proliferative sickle cell retinopathy. Br J Ophthalmol.

[7] Rodrigues M, Kashiwabuchi F, Deshpande M (2016). Expression pattern of HIF-1α and VEGF supports circumferential application of scatter laser for proliferative sickle retinopathy. Invest Ophthalmol Vis Sci.

[8] Dembélé AK, Toure BA, Sarro YS (2017). Prévalence et facteurs de risqué de la rétinopathie drépanocytaire dans un centre de suivi drépanocytaire d’Afrique subsaharienne. [Prevalence and risk factors for sickle retinopathy in a sub-Saharan comprehensive Sickle Cell Center]. Rev Med Interne..

[9] Diallo JW, Sanfo O, Blot I (2009). Étude épidémiologique et facteurs pronostiques de la rétinopathie drépanocytaire à Ouagadougou (Burkina Faso). [Epidemiology and prognostic factors for SCR in Ouagadougou (Burkina Faso)]. J Fr Ophtalmol..

[10] Downes SM, Hambleton IR, Chuang EL, Lois N, Serjeant GR, Bird AC (2005). Incidence and natural history of proliferative sickle cell retinopathy: observations from a cohort study. Ophthalmology.

[11] Fox PD, Dunn DT, Morris JS, Serjeant GR (1990). Risk factors for proliferative sickle retinopathy. Br J Ophthalmol.

[12] Leveziel N, Bastuji-Garin S, Lalloum F (2011). Clinical and laboratory factors associated with the severity of proliferative SCR in patients with sickle cell hemoglobin C (SC) and homozygous sickle cell (SS) disease. Medicine (Baltimore)..

[13] Yawn BP, Buchanan GR, Afenyi-Annan AN (2014). Management of sickle cell disease: summary of the 2014 evidence-based report by expert panel members. JAMA.

[14] Klufas MA, Kiss S, Ryan SJ, Schachat AP, Wilkinson CP, Hinton DR, Sadda SR, Wiedemann P (2017). Widefield imaging. Ryan retina 6e.

[15] Early Treatment Diabetic Retinopathy Study (ETDRS) Research Group. Fundus photographic risk factors for progression of diabetic retinopathy. ETDRS report number 12. Ophthalmology. 1991;98:823–33. 2062515

[16] Goldberg MF (1971). Classification and pathogenesis of proliferative sickle retinopathy. Am J Ophthalmol.

[17] Penman AD, Talbot JF, Chuang EL, Thomas P, Serjeant GR, Bird AC (1994). New classification of peripheral retinal vascular changes in sickle cell disease. Br J Ophthalmol.

[18] Chow CC, Genead MA, Anastasakis A, Chau FY, Fishman GA, Lim JI (2011). Structural and functional correlation in sickle cell retinopathy using spectral-domain optical coherence tomography and scanning laser ophthalmoscope microperimetry. Am J Ophthalmol.

[19] Han IC, Tadarati M, Scott AW (2015). Macular vascular abnormalities identified by optical coherence tomographic angiography in patients with Sickle Cell Disease. JAMA Ophthalmol..

[20] Han IC, Tadarati M, Pacheco KD, Scott AW (2017). Evaluation of macular vascular abnormalities identified by optical coherence tomography angiography in sickle cell disease. Am J Ophthalmol.

[21] Prasad PS, Oliver SCN, Coffee RE (2010). Ultra wide-field angiographic characteristics of branch retinal and hemicentral retinal vein occlusion. Ophthalmology.

[22] Tsui I, Franco-Cardenas V, Hubschman J-P (2012). Ultra wide field fluorescein angiography can detect macular pathology in central retinal vein occlusion. Ophthalmic Surg Lasers Imaging..

[23] Patel CK, Fung TH, Muqit MM (2013). Non-contact ultra-widefield imaging of retinopathy of prematurity using the Optos dual wavelength scanning laser ophthalmoscope. Eye (Lond)..

[24] Wessel MW, Aaker GD, Parlitsis G (2012). Ultra-wide-field angiography improves the detection and classification of diabetic retinopathy. Retina..

[25] Reddy S, Shwartz SD (2009). Ultra wide field fluorescein angiography guided targeted retinal photocoagulation. Semin Ophthalmol..

[26] Cho M, Kiss S (2011). Detection and monitoring of sickle cell retinopathy using ultra wide-field color photography and fluorescein angiography. Retina..

[27] Han IC, Zhang AY, Liu TYA, Linz MO, Scott AW (2018). Utility of ultra-widefield retinal imaging for the staging and management of sickle cell retinopathy. Retina..

[28] Pahl DA, Green NS, Bhatia M (2017). Optical coherence tomography angiography and ultra-widefield fluorescein angiography for early detection of adolescent Sickle Retinopathy. Am J Ophthalmol.

[29] Croft DE, van Hemert J, Wykoff CC (2014). Precise montaging and metric quantification of retinal surface area from ultra-widefield fundus photography and fluorescein angiography. Ophthalmic Surg Lasers Imaging Retina..

[30] Han IC, Linz MO, Liu TYA, Zhang AY, Tian J, Scott AW (2017). Correlation of ultra-widefield fluorescein angiography and OCT angiography in sickle cell retinopathy. Ophthalmol Retina..

[31] Ghasemi Falavarjani K, Scott AW, Wang K (2016). Correlation of multimodal imaging in sickle cell retinopathy. Retina..

[32] Witmer MT, Kiss S (2013). Wide-field imaging of the retina. Surv Ophthalmol.

[33] Hood MP, Diaz RI, Sigler EJ, Calzada JI (2016). Temporal macular atrophy as a predictor of neovascularization in sickle cell retinopathy. Ophthalmic Surg Lasers Imaging Retina..

[34] Myint KT, Sahoo S, Thein AW, Moe S, Ni H. Laser therapy for retinopathy in sickle cell disease. Cochrane Database Syst Rev. 2015;10:CD010790. 10.1002/14651858.CD010790.pub2 PMC8741205 26451693

[35] Piel FB, Hay SI, Gupta S, Weatherall DJ, Williams TN (2013). Global burden of sickle cell anaemia in children under five, 2010–2050: modelling based on demographics, excess mortality, and interventions. PLoS Med..

[36] Adhi M, Silva FQ, Lang R (2017). Non-mydriatic ultra-widefield imaging compared with single-field imaging in the evaluation of peripheral retinal pathology. Ophthalmic Surg Lasers Imaging Retina..

[37] Ting DSW, Cheung CY, Lim G (2017). Development and validation of a deep learning system for diabetic retinopathy and related eye diseases using retinal images from multiethnic populations with diabetes. JAMA.

